# Supplementary material for: Meta-Analysis and Gene Set Enrichment Relative to ER Status Reveal Elevated Activity of MYC and E2F in the “Basal” Breast Cancer Subgroup
Source: PLoS One. 2009 Mar 9;4(3):e4710. doi: 10.1371/journal.pone.0004710 (PMC2650420; doi:10.1371/journal.pone.0004710)
Supplement: Dataset S1 — GSEA results from first screen. The file contains a zipped archive of the HTML results of the second GSEA screen. Reports for enriched or depleted datasets may be accessed from the “index.html” link within the my_analysis.GseaPreranked.1219975950625.rpt.zip ZIP archive. Enrichment plots are available for each of the 50 most enriched and depleted gene sets. (13.32 MB ZIP) [file pone.0004710.s001.zip › my_analysis.GseaPreranked.1219975950625.rpt.zip!//LEE_TCELLS3_UP.html]

Details for gene set LEE\_TCELLS3\_UP[GSEA]

|  || Dataset | zSco.ERneg.to.ERpos\_collapsed |
| Phenotype | NoPhenotypeAvailable |
| Upregulated in class | na\_pos |
| GeneSet | LEE\_TCELLS3\_UP |
| Enrichment Score (ES) | 0.69401425 |
| Normalized Enrichment Score (NES) | 2.8180077 |
| Nominal p-value | 0.0 |
| FDR q-value | 0.0 |
| FWER p-Value | 0.0 |
Table: GSEA Results Summary

  

Fig 1: Enrichment plot: LEE\_TCELLS3\_UP      
 Profile of the Running ES Score & Positions of GeneSet Members on the Rank Ordered List

  

| PROBE | GENE SYMBOL | GENE\_TITLE | RANK IN GENE LIST | RANK METRIC SCORE | RUNNING ES | CORE ENRICHMENT || 1 | TTK |  |  | 11 | 7.059 | 0.0225 | Yes |
| 2 | NCAPG |  |  | 29 | 6.628 | 0.0431 | Yes |
| 3 | BUB1 |  |  | 41 | 6.445 | 0.0636 | Yes |
| 4 | TMSL8 |  |  | 50 | 6.319 | 0.0839 | Yes |
| 5 | CENPA |  |  | 51 | 6.315 | 0.1048 | Yes |
| 6 | MKI67 |  |  | 98 | 5.871 | 0.1208 | Yes |
| 7 | KIF2C |  |  | 124 | 5.703 | 0.1377 | Yes |
| 8 | GGH |  |  | 161 | 5.504 | 0.1532 | Yes |
| 9 | MCM4 |  |  | 180 | 5.436 | 0.1698 | Yes |
| 10 | MELK |  |  | 181 | 5.424 | 0.1878 | Yes |
| 11 | MCM10 |  |  | 201 | 5.315 | 0.2039 | Yes |
| 12 | KIF14 |  |  | 221 | 5.215 | 0.2197 | Yes |
| 13 | PTTG1 |  |  | 225 | 5.206 | 0.2367 | Yes |
| 14 | BIRC5 |  |  | 229 | 5.191 | 0.2536 | Yes |
| 15 | CKS2 |  |  | 239 | 5.145 | 0.2700 | Yes |
| 16 | KNTC2 |  |  | 256 | 5.074 | 0.2856 | Yes |
| 17 | RAD51AP1 |  |  | 267 | 5.028 | 0.3014 | Yes |
| 18 | BUB1B |  |  | 271 | 5.016 | 0.3178 | Yes |
| 19 | CEP55 |  |  | 279 | 4.995 | 0.3338 | Yes |
| 20 | CDC25A |  |  | 281 | 4.993 | 0.3502 | Yes |
| 21 | LOC146909 |  |  | 317 | 4.875 | 0.3637 | Yes |
| 22 | KIF20A |  |  | 318 | 4.874 | 0.3798 | Yes |
| 23 | OIP5 |  |  | 320 | 4.868 | 0.3958 | Yes |
| 24 | CDCA3 |  |  | 321 | 4.868 | 0.4119 | Yes |
| 25 | ASPM |  |  | 359 | 4.767 | 0.4249 | Yes |
| 26 | CCNB2 |  |  | 431 | 4.589 | 0.4347 | Yes |
| 27 | AURKA |  |  | 460 | 4.483 | 0.4474 | Yes |
| 28 | TNFRSF21 |  |  | 504 | 4.342 | 0.4585 | Yes |
| 29 | ADA |  |  | 510 | 4.330 | 0.4725 | Yes |
| 30 | DLG7 |  |  | 521 | 4.306 | 0.4860 | Yes |
| 31 | PRC1 |  |  | 543 | 4.253 | 0.4984 | Yes |
| 32 | DSCR1 |  |  | 555 | 4.225 | 0.5116 | Yes |
| 33 | DCC1 |  |  | 567 | 4.206 | 0.5247 | Yes |
| 34 | TUBB |  |  | 582 | 4.183 | 0.5374 | Yes |
| 35 | TFDP2 |  |  | 589 | 4.174 | 0.5508 | Yes |
| 36 | AKAP2 /// PALM2-AKAP |  |  | 599 | 4.155 | 0.5639 | Yes |
| 37 | MCM2 |  |  | 630 | 4.080 | 0.5751 | Yes |
| 38 | TSHR |  |  | 703 | 3.923 | 0.5826 | Yes |
| 39 | KIF11 |  |  | 713 | 3.909 | 0.5949 | Yes |
| 40 | TYMS |  |  | 829 | 3.738 | 0.5985 | Yes |
| 41 | NUSAP1 |  |  | 836 | 3.732 | 0.6104 | Yes |
| 42 | GINS1 |  |  | 902 | 3.632 | 0.6175 | Yes |
| 43 | UBE2C |  |  | 994 | 3.489 | 0.6222 | Yes |
| 44 | E2F8 |  |  | 1122 | 3.334 | 0.6236 | Yes |
| 45 | HMGB3 |  |  | 1128 | 3.330 | 0.6342 | Yes |
| 46 | CDC2 |  |  | 1259 | 3.182 | 0.6349 | Yes |
| 47 | CD1A |  |  | 1284 | 3.154 | 0.6435 | Yes |
| 48 | KIF15 |  |  | 1361 | 3.068 | 0.6479 | Yes |
| 49 | HELLS |  |  | 1382 | 3.057 | 0.6565 | Yes |
| 50 | ATAD2 |  |  | 1532 | 2.917 | 0.6549 | Yes |
| 51 | HMMR |  |  | 1608 | 2.843 | 0.6587 | Yes |
| 52 | CCNB1 |  |  | 1626 | 2.823 | 0.6667 | Yes |
| 53 | NEK2 |  |  | 1703 | 2.758 | 0.6701 | Yes |
| 54 | KIAA1794 |  |  | 1724 | 2.747 | 0.6777 | Yes |
| 55 | CDKN3 |  |  | 1827 | 2.674 | 0.6788 | Yes |
| 56 | PBK |  |  | 1853 | 2.647 | 0.6857 | Yes |
| 57 | SYK |  |  | 1859 | 2.641 | 0.6940 | Yes |
| 58 | RRM2 |  |  | 2047 | 2.485 | 0.6881 | No |
| 59 | MPP1 |  |  | 2289 | 2.329 | 0.6776 | No |
| 60 | RAPGEF5 |  |  | 3072 | 1.896 | 0.6247 | No |
| 61 | FAIM |  |  | 3391 | 1.753 | 0.6065 | No |
| 62 | NEIL3 |  |  | 3412 | 1.743 | 0.6107 | No |
| 63 | TOP2A |  |  | 3735 | 1.610 | 0.5917 | No |
| 64 | TUBB2C |  |  | 3736 | 1.610 | 0.5971 | No |
| 65 | CPVL |  |  | 3832 | 1.578 | 0.5951 | No |
| 66 | DNTT |  |  | 3841 | 1.574 | 0.5997 | No |
| 67 | CD1B |  |  | 4036 | 1.499 | 0.5900 | No |
| 68 | GNA15 |  |  | 4144 | 1.455 | 0.5867 | No |
| 69 | NLGN4X |  |  | 4375 | 1.365 | 0.5738 | No |
| 70 | DTL |  |  | 4509 | 1.314 | 0.5681 | No |
| 71 | WHSC1 |  |  | 4717 | 1.247 | 0.5566 | No |
| 72 | KIAA0101 |  |  | 4770 | 1.230 | 0.5567 | No |
| 73 | E2F2 |  |  | 5382 | 1.019 | 0.5139 | No |
| 74 | GFI1 |  |  | 5571 | 0.951 | 0.5028 | No |
| 75 | PON1 |  |  | 5679 | 0.921 | 0.4978 | No |
| 76 | MLF1IP |  |  | 5991 | 0.815 | 0.4770 | No |
| 77 | ARPP-21 |  |  | 6335 | 0.706 | 0.4534 | No |
| 78 | LETM1 |  |  | 6363 | 0.697 | 0.4537 | No |
| 79 | PACAP |  |  | 7358 | 0.365 | 0.3797 | No |
| 80 | CASC5 |  |  | 7587 | 0.273 | 0.3634 | No |
| 81 | DIAPH3 |  |  | 7628 | 0.259 | 0.3612 | No |
| 82 | CD1E |  |  | 8121 | 0.065 | 0.3242 | No |
| 83 | RAG1 |  |  | 8450 | -0.056 | 0.2996 | No |
| 84 | CEP70 |  |  | 8602 | -0.131 | 0.2887 | No |
| 85 | PALLD |  |  | 8851 | -0.232 | 0.2707 | No |
| 86 | CD99 |  |  | 9887 | -0.776 | 0.1950 | No |
| 87 | SCRN1 |  |  | 9991 | -0.836 | 0.1900 | No |
| 88 | C18ORF1 |  |  | 10918 | -1.494 | 0.1249 | No |
| 89 | SLC1A4 |  |  | 12163 | -2.796 | 0.0401 | No |
| 90 | MYB |  |  | 12539 | -3.318 | 0.0227 | No |
| 91 | AEBP1 |  |  | 12698 | -3.612 | 0.0227 | No |
| 92 | GALNT7 |  |  | 12708 | -3.629 | 0.0341 | No |
| 93 | KIAA0182 |  |  | 12724 | -3.667 | 0.0451 | No |
Table: GSEA details [plain text format]

  

Fig 2: LEE\_TCELLS3\_UP: Random ES distribution      
 Gene set null distribution of ES for **LEE\_TCELLS3\_UP**
